# Similar brain proteomic signatures in Alzheimer’s disease and epilepsy

**DOI:** 10.1007/s00401-024-02683-4

**Published:** 2024-01-30

**Authors:** Dominique Leitner, Geoffrey Pires, Tomas Kavanagh, Evgeny Kanshin, Manor Askenazi, Beatrix Ueberheide, Orrin Devinsky, Thomas Wisniewski, Eleanor Drummond

**Affiliations:** 1 Center for Cognitive Neurology, Department of Neurology, Grossman School of Medicine, New York University, New York, NY 10016 USA; 2 Brain and Mind Centre and School of Medical Sciences, University of Sydney, Camperdown, NSW 2050 Australia; 3 Proteomics Laboratory, Division of Advanced Research Technologies and Department of Biochemistry and Molecular Pharmacology, New York University Grossman School of Medicine, New York, 10016 USA; 4 Biomedical Hosting LLC, Arlington, MA 02140 USA; 5 Comprehensive Epilepsy Center, New York University Grossman School of Medicine, New York, 10016 USA

**Keywords:** Alzheimer’s disease, Epilepsy, Proteomics, Tau, Mass spectrometry, Beta amyloid

## Abstract

The prevalence of epilepsy is increased among Alzheimer’s Disease (AD) patients and cognitive impairment is common among people with epilepsy. Epilepsy and AD are linked but the shared pathophysiological changes remain poorly defined. We aim to identify protein differences associated with epilepsy and AD using published proteomics datasets. We observed a highly significant overlap in protein differences in epilepsy and AD: 89% (689/777) of proteins altered in the hippocampus of epilepsy patients were significantly altered in advanced AD. Of the proteins altered in both epilepsy and AD, 340 were altered in the same direction, while 216 proteins were altered in the opposite direction. Synapse and mitochondrial proteins were markedly decreased in epilepsy and AD, suggesting common disease mechanisms. In contrast, ribosome proteins were increased in epilepsy but decreased in AD. Notably, many of the proteins altered in epilepsy interact with tau or are regulated by tau expression. This suggests that tau likely mediates common protein changes in epilepsy and AD. Immunohistochemistry for Aβ and multiple phosphorylated tau species (pTau396/404, pTau217, pTau231) showed a trend for increased intraneuronal pTau217 and pTau231 but no phosphorylated tau aggregates or amyloid plaques in epilepsy hippocampal sections. Our results provide insights into common mechanisms in epilepsy and AD and highlights the potential role of tau in mediating common pathological protein changes in epilepsy and AD.

**Supplementary Information:**

The online version contains supplementary material available at 10.1007/s00401-024-02683-4.

## Introduction

There is a bidirectional relationship between epilepsy and Alzheimer’s disease (AD): people with epilepsy are more likely to develop AD and people with AD are more likely to have seizures. People with epilepsy have significantly faster age-associated cognitive decline than controls [5] and a twofold increased dementia risk compared to controls [49]. This increases to a threefold increased dementia risk if they have late-onset epilepsy [22]. Among AD patients, 10–22% have unprovoked seizures [60] and 22–54% of AD patients have subclinical epileptiform activity [18, 19, 25, 59]. Seizures in AD are associated with accelerated cognitive decline [57], more pronounced differences in CSF AD biomarkers [3], and greater AD neuropathology (e.g., decreased brain weight, increased phosphorylated tau [pTau], increased white matter beta amyloid [Aβ] plaque pathology) [13]. An anti-seizure medication attenuates cognitive impairment in AD mouse models and improves executive function and spatial memory in AD patients with seizures or subclinical epileptiform activity [58].

People with epilepsy have greater AD-associated pathology than age-matched controls: epilepsy cases, particularly temporal lobe epilepsy (TLE) cases, have more pTau aggregates than controls [12, 40, 48, 53, 54]. pTau aggregate morphologies in epilepsy vary, but overlap with AD and chronic traumatic encephalopathy (e.g., neurofibrillary tangles [NFTs], neuronal pre-tangles, neuropil threads, axonal staining, extracellular deposits, subpial band staining, and astrocytic tau pathology). However, questions remain about the prevalence of epilepsy-associated pTau pathology in epilepsy, as it has not been observed in all studies [45]. The amount of pTau pathology in epilepsy correlates with cognitive decline [52], in some but not all studies [53, 54], implicating other factors. Aβ plaques occur in some epilepsy patients [12, 30, 45, 52, 53], but are typically sparse and variable across studies [54]. Aβ pathology correlates with age [30], suggesting it is an age-related feature rather than a mechanistic factor. Aβ pathology did not correlate with cognitive decline in epilepsy [12]. Thus, tau pathology is common in epilepsy and more prevalent than Aβ pathology, suggesting that some epilepsies may be tauopathies [54].

The role of tau in epilepsy is supported by experimental studies. Tau overexpression in mouse models commonly cause seizures [60]. Reducing tau in epilepsy mouse models (particularly in excitatory neurons) is therapeutic: it decreases mortality, seizures, the overactivation of the mTOR signalling pathway, and ameliorates abnormalities in learning and memory [11, 44]. Furthermore, enhancing levels of a specific type of pTau shown to be protective in AD (pTau205) also proved protective in mouse models of epilepsy, decreasing seizures, behavioural deficits and mortality [34].

Despite convincing evidence supporting an interaction between epilepsy and AD, knowledge of the underlying mechanisms is limited. While pTau has emerged as a potential link between epilepsy and AD, further studies are needed to explore the common molecular mechanisms involved. The aim of this study was to determine if there were common protein differences in the brain in epilepsy and AD using the most comprehensive publicly available proteomic datasets for each disease [1, 39]. Based on the previous literature, we hypothesized that there would be many common protein differences in epilepsy and AD, particularly in proteins associated with tau pathology.

## Methods

### Proteomic comparison of proteins altered in epilepsy and AD

Proteins significantly altered in human epilepsy brain tissue were obtained from our previously published study [39]. In this study, epilepsy cases were acquired through the North American SUDEP Registry and included cases from multiple sites around the country whose cause of death was not SUDEP. This cohort encompassed a broad representation of various epilepsies in the cases with known clinical information, the majority of which excluded known frontal lobe involvement [39]. The proteomic dataset obtained from this previous study consisted of 777 proteins significantly altered in the microdissected CA1-3 hippocampal region in epilepsy versus controls based on a false discovery rate of < 5% (Supplementary Table 1). The microdissected tissue included any neuropathological lesions present in CA1-3. While this previous study also documented protein changes in the frontal cortex and dentate gyrus in epilepsy, we used protein changes in the hippocampus only as the representative dataset of protein changes in epilepsy because this region had the most extensive disease-associated changes in epilepsy. Comparison between frontal cortex and dentate gyrus-specific protein changes in epilepsy and AD is detailed in Supplementary Table 14.

Proteins altered in AD were obtained from NeuroPro (v1.12; https://neuropro.biomedical.hosting/) [1]. NeuroPro is a combined analysis of protein differences in human AD brain tissue identified in 38 published proteomic studies. At the time of use (May 2023), NeuroPro included 5,311 proteins altered in human AD brain tissue, detailing significant protein differences across 13 brain regions, 3 clinical stages of AD (preclinical AD, mild cognitive impairment and advanced AD) and proteins associated with AD neuropathological lesions (plaques, NFTs, cerebral amyloid angiopathy [CAA]). For this study, NeuroPro was filtered to identify proteins (a) altered in bulk tissue homogenate in advanced AD in comparison to controls (Supplementary Table 2) and (b) enriched in AD neuropathological lesions (amyloid plaques, NFTs, CAA) in comparison to corresponding neighbouring non-lesion tissue (Supplementary Table 3). Proteins that were present (but not enriched) in neuropathological lesions were not included in this analysis. For proteins altered in advanced AD bulk tissue, proteins were considered increased/decreased in AD if they were consistently reported as altered in the same direction in all studies included in NeuroPro. Consistently altered proteins received a score (“NeuroPro Score”) based on a count of the number of times this protein was reported significantly altered in advanced AD in bulk tissue in previous studies. Proteins consistently decreased in previous studies received a negative score and proteins consistently increased in previous studies received a positive score. One inconsistent directional change was permitted for proteins with a score > 5. All remaining proteins with inconsistent directional changes were designated “Inconsistent” and did not receive a score.

Phosphorylated tau interactors were obtained from [7]. In this study, pTau interactors were identified using co-immunoprecipitation of PHF1-immunoreactive pTau from human advanced AD brain tissue. LC–MS identified 125 protein interactors of PHF1-immunoreactive pTau using a probabilistic SAINT score that defined proteins that interacted more significantly with pTau than control IgG (Supplementary Table 4). Total tau interactors were obtained from [20]. Total tau interactors were identified by co-immunoprecipitation of Tau5-immunoreactive total tau from human advanced AD and control brain tissue followed by LC–MS. To allow for comparison with other datasets, the published dataset was stripped of isoforms and all duplicates were removed, resulting in identification of 511 proteins that significantly interacted with total tau in AD brain tissue in comparison to control brain tissue (significance defined by paired t-test p < 0.05 and fold-change > 1.5) (Supplementary Table 5).

Protein datasets were matched using UniProt ID or Gene ID. A match of either identifier was sufficient to account for mapping difficulties where multiple Uniprot IDs map to the same gene ID and vice versa. All proteomic data were obtained using LC–MS, a bottom-up proteomics approach.

### Upstream regulators of proteins significantly altered in epilepsy

Ingenuity Pathway Analysis (Qiagen) was used to identify upstream regulator proteins (accessed May 15th 2023; version: 90,348,151; Supplementary Table 6). Upstream regulator output was limited to “Genes, RNAs, & Proteins” to identify endogenous regulators of epilepsy protein differences.

### Comparison with causative epilepsy genes

Genes causally linked to epilepsy were defined as those with either “strong” or “definitive” classification from the ClinGen Epilepsy Expert Panel (accessed 4/5/23). https://search.clinicalgenome.org/kb/affiliate/10005?page=1&size=25&search = (complete list available in Supplementary Table 7).

### Bioinformatics and pathway analysis

Heatmaps were generated using epilepsy data [39]. Significant hippocampal proteins that were also reported as altered in AD in NeuroPro were evaluated by unsupervised hierarchical clustering and represented by a heatmap with R package *ComplexHeatmap*, by kmeans clustering. Enrichment stats were calculated in R using a Fisher’s exact test in the GeneOverlap v1.36.0 package. Gene ontology over-enrichment analysis was performed in R with the clusterProfiler v4.8.1 package using the human database org.Hs.eg.db v3.17.0 package. Background was set to the total identified subset of genes in Neuropro (5236 genes in the annotation). Outputs were filtered to a q-value of 0.05 and top ten pathways are plotted (where available). Subsets used included proteins up- and down-regulated in both epilepsy and AD, and proteins changed in the opposite direction in epilepsy and AD. STRING v12.0 [50] was used to create a full STRING network of protein–protein interactions for proteins up- or down-regulated in both epilepsy and AD with the cut-off set to high. STRING v12.0 was also used to annotate protein groups for altered epilepsy proteins that are known phosphorylated tau interactors with the gene ontology: cellular compartment annotation set. Interaction networks were annotated in Cytoscape v3.10.0. All figures were edited in Adobe Illustrator v27.8 and selected figure panels were created with BioRender.com. Synaptic proteins were defined using SynGO (Release 1.1; [24]). Mitochondrial proteins and their primary functions were defined using MitoCoP [35].

### Human brain tissue

Human brain tissue specimens were acquired under protocols with Institutional Review Board (IRB) approval at NYU Grossman School of Medicine, including autopsy tissues from the North American SUDEP Registry (NASR) at NYU CEC and NYU Center for Biospecimen Research and Development (CBRD)/Department of Pathology. The same cases (control *n* = 14, epilepsy *n* = 13) evaluated in our previous proteomics study [39] were evaluated by immunohistochemistry in the current study. Case history is summarized in Table 1.

**Table 1:** Patient information

Case ID	Age	Sex	PMI	Significant neuropathology	Disease duration	Aβ (4G8) IHC	PHF1 IHC	pTau217 IHC	pTau231 IHC
Control 1	55	M	64	None		Yes	Yes	Yes	Yes
Control 2	57	M	142	None		Yes	Yes	NA	NA
Control 3	56	F	46	None		Yes	Yes	Yes	Yes
Control 4	49	F	144	None		Yes	Yes	NA	NA
Control 5	59	F	57	None		Yes	Yes	Yes	Yes
Control 6	59	M	116	None		Yes	Yes	Yes	Yes
Control 7	57	M	91	None		Yes	Yes	Yes	Yes
Control 8	55	M	57	Subacute, right temporal white matter microinfarct		Yes	Yes	Yes	Yes
Control 9	59	M	60	None		Yes	Yes	Yes	Yes
Control 10	38	M	44	Acute and chronic hypoxic-ischemic changes, diffuse		Yes	Yes	NA	NA
Control 11	59	M	< 120	Ischemic infarct, left frontal MCA/ACA		Only cortex	Only cortex	NA	NA
Control 12	49	M	48	Meningeal fibrosis		Yes	Yes	Yes	Yes
Control 13	50	M	50	Hypoxic-ischemic changes, diffuse		Yes	Yes	Yes	NA
Control 14	54	M	66	Hypoxic-ischemic injury, acute, multifocal		Yes	Yes	Yes	Yes
Epilepsy 1	36	M	20	None	8	Yes	Yes	Yes	Yes
Epilepsy 2	54	M	< 24	None	1	Yes	Yes	Yes	NA
Epilepsy 3	64	F	18	None	ND	Yes	Yes	NA	NA
Epilepsy 4	9	F	30	FCD	8	Yes	Yes	Yes	Yes
Epilepsy 5	45	M	27	DGD	20	Yes	Yes	Yes	Yes
Epilepsy 6	36	M	48	Sclerosis	12	Yes	Yes	Yes	Yes
Epilepsy 7	45	M	< 48	None	43	Yes	Yes	Yes	Yes
Epilepsy 8	24	F	< 48	DGD	ND	Yes	Yes	Yes	Yes
Epilepsy 9	28	M	< 48	DGD	22	Yes	Yes	Yes	NA
Epilepsy 10	23	M	< 48	FCD	ND	Yes	Yes	Yes	Yes
Epilepsy 11	34	F	13	FCD	32	Yes	Yes	Yes	Yes
Epilepsy 12	32	M	19	None	10	Yes	Yes	Yes	Yes
Epilepsy 13	49	M	43	DGD, sclerosis	48.4	Yes	Yes	Yes	Yes

*DGD*  dentate gyrus dysgenesis [26], *FCD*  focal cortical dysplasia, *HS*  hippocampal sclerosis, *IHC*  immunohistochemistry, *MCA/ACA*  middle cerebral artery/anterior cerebral artery, *ND*  not determined, *NA*  not available from hippocampal section, *PMI*  post mortem interval

### Immunohistochemistry

For the majority of cases, Aβ and PHF1 (pTau396/404) immunohistochemistry were performed as part of neuropathology analyses at the NYU CBRD Core. Briefly, formalin-fixed, paraffin embedded (FFPE) tissues were sectioned at 8 μm. Slides were dried in a 60 °C incubator for 1 h, deparaffinized in xylenes, rehydrated through graded alcohols, and rinsed in distilled water. Sections were treated with 88% formic acid for 7 min at room temperature followed by washing in running distilled water for 5 min. Chromogenic IHC was performed on a Ventana Medical Systems Discovery Ultra platform using Ventana reagents except as noted. Antigen retrieval was performed in Ultra Cell Conditioner 2 (Ventana Medical Systems Cat# 950-223) for 24 min at 91 °C. Endogenous peroxidase activity was blocked with hydrogen peroxide for 4 min. Primary antibodies against Aβ (4G8, Biolegend #800,711, 1:1000) or PHF1 (provided by Peter Davies [14], 1:1600) were diluted in antibody diluent (Ventana Medical Systems Cat# ADB250) and incubated for 2 h at 37 °C. Antibodies were detected using Ultraview Universal horseradish peroxidase conjugated goat anti-mouse multimer followed by diaminobenzidine (DAB) substrate (Ventana Medical Systems Cat# 760–500). Slides were washed in distilled water, counterstained with hematoxylin, dehydrated, and mounted with permanent media.

For the remaining cases, 8 μm sections were deparaffinized and rehydrated through a series of xylenes and ethanol, followed by antigen retrieval with 88% formic acid, and 10 mM sodium citrate with 0.05% Triton-X 100 at pH 6. Sections were blocked in 10% normal goat serum for 1 h, and incubated in 4G8 (Biolegend #800,711, 1:1000), PHF1 (provided by Peter Davies [14], 1:200), pTau217 (ThermoFisher #44–744, 1:250), or pTau231 (AT180; ThermoFisher #MN1040, 1:250) primary antibodies overnight at 4˚C. After washing, sections were incubated in biotinylated mouse secondary antibody (Vector Laboratories, 1:1000) for 1 h, followed by avidin–biotin peroxidase (Vector Laboratories) for 30 min. After washing, sections were incubated with DAB chromogen solution (ThermoFisher) and counterstained with hematoxylin. With additional washes, sections were coverslipped. Sections were included in semi-quantitation or quantitation if they contained CA1-3 and staining was successful. This resulted in exclusion of a small number of cases that was specific to each staining run (Table 1).

### IHC Aβ and PHF1 semi-quantitation

Aβ and PHF1 were semi-quantitated from whole slide scanned images of hippocampal sections. Whole slide scanning was performed at 20X magnification with a Leica Aperio Versa 8 microscope. A regional Aβ or PHF1 score (0, 1, 2, 3) was given for each slide to indicate relative level of pathology.

### IHC pTau quantitation

pTau217 and pTau231 were quantified from whole slide scanned images of hippocampal sections. Whole slide scanning was performed at 20X magnification with a Leica Aperio Versa 8 microscope. The percent positive pixel area in CA1-3 was calculated using Leica Aperio ImageScope. The “Positive Pixel Count” algorithm was used with modification to color saturation threshold = 0 and Iwp(High) = 80. The number of positive pixels was then calculated from the total pixels in an area, performed in each ROI of interest (CA1, CA2, CA3) which was then averaged across all regions per case. To evaluate clustering by clinical variables of interest, quantified histology positive area was further evaluated by unsupervised hierarchical clustering and represented by a heatmap with R package *ComplexHeatmap*.

### WGCNA

Weighted gene correlation network analysis (WGCNA) was performed to determine how proteins measured by proteomics corresponded to pTau levels quantified from immunohistochemistry, and to identify the functional annotations of protein clusters identified. WGCNA was performed in the R environment with the *WGCNA* package for blockwiseModules with defaults except where noted, similar to previously described [21, 27, 28, 43]. Soft threshold power beta was determined at R^2^ = 0.8 (power = 4). Gene ontology annotations for modules was determined following WGCNA with the *anRichment* package in the R environment with Entrez IDs against the human GOcollection. GO biological process annotations were considered with an FDR < 5% and associated with at least 5 proteins (Supplementary Table 15).

## Results

### Similar protein differences in epilepsy and AD

**Fig. 1:**
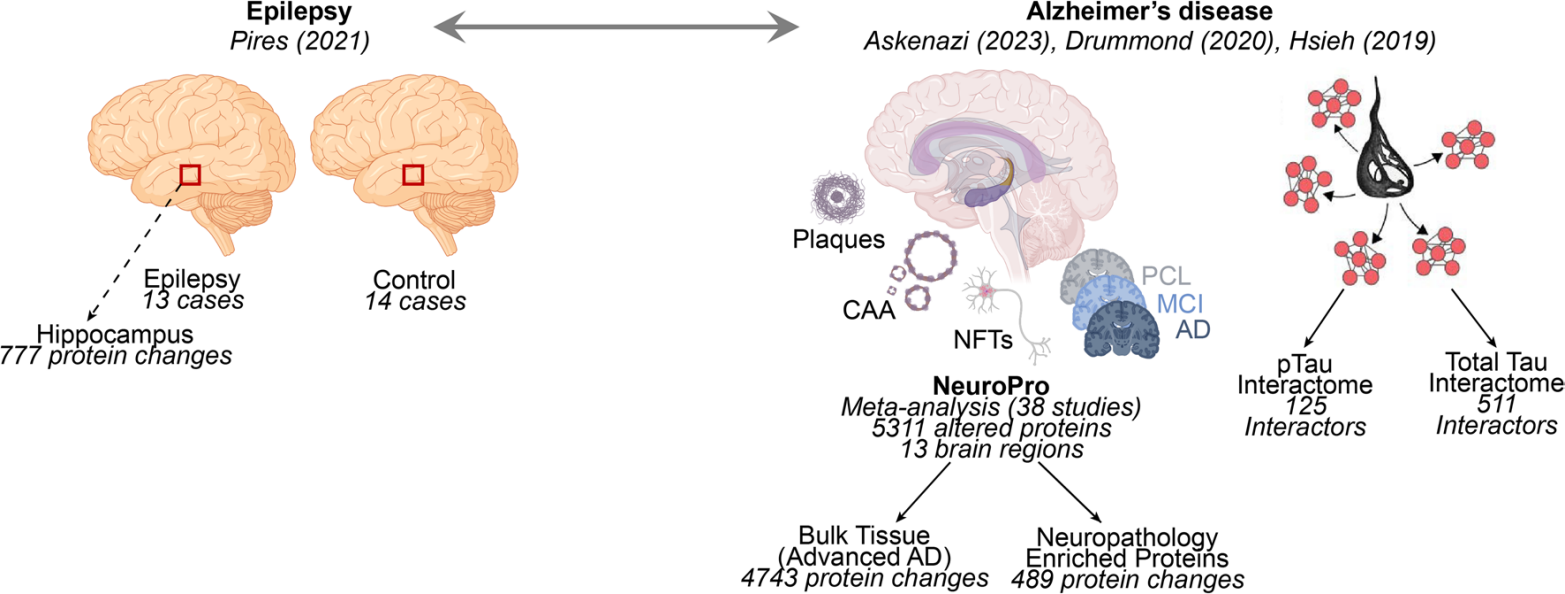
Methods schematic. A proteomic dataset of protein changes in human brain tissue from individuals with epilepsy was obtained from Pires et al*.* [39]. This dataset identified 777 significantly altered proteins in the hippocampus from individuals with epilepsy. This dataset was compared to three proteomic datasets detailing protein changes in AD: (1) a meta-analysis detailing all protein changes in human AD brain tissue (NeuroPro; [1]. Two subsets of this dataset were used in this study – 4743 protein changes reported in advanced AD brain tissue and 489 AD neuropathology-enriched proteins. (2) a dataset detailing 125 proteins that interact with phosphorylated tau in human AD brain tissue [7] and (3) a dataset detailing 511 proteins that interact with total tau in human AD brain tissue [20]

**Fig. 2:**
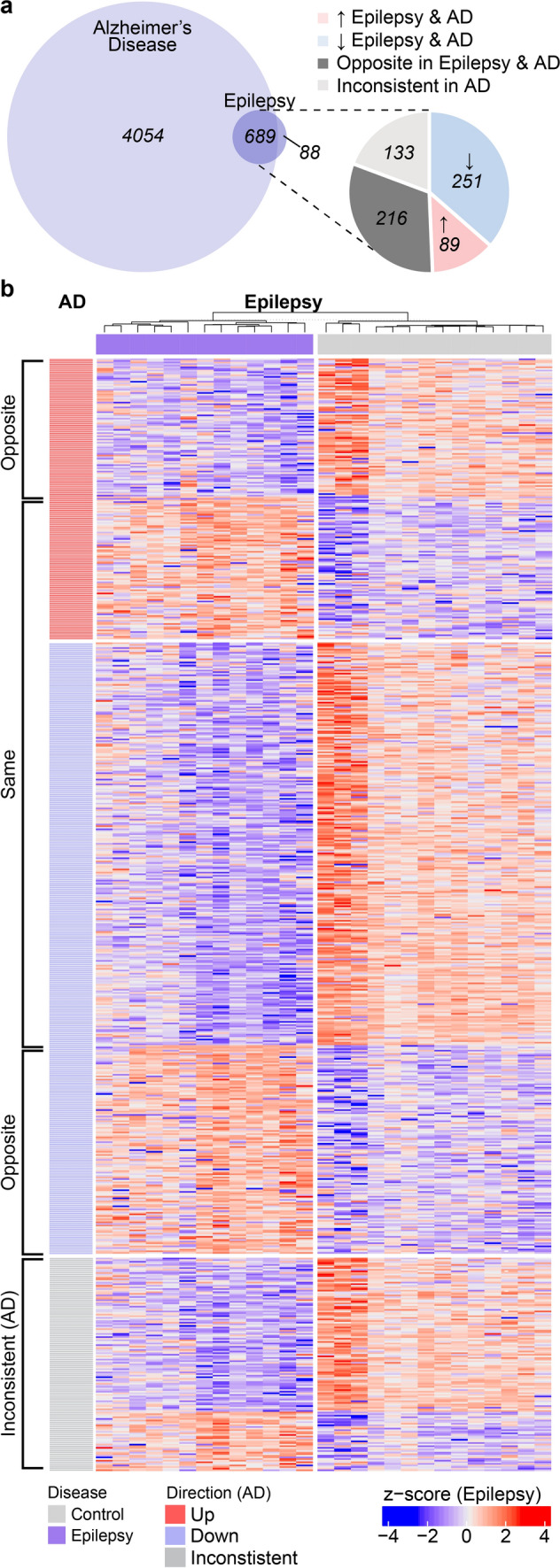
Comparison of protein changes in epilepsy and AD. **a** Comparison of protein changes in epilepsy hippocampus and protein changes in advanced AD human brain tissue revealed 89% protein differences in epilepsy (689/777 protein changes) were also significantly altered in advanced AD. Pie chart shows proportion of protein differences that were consistently altered in the same direction in both disease groups, proteins altered in the opposite direction in epilepsy and AD, and proteins that were unable to be annotated as increased/decreased in AD due to inconsistencies between studies included in the meta-analysis. **b** Heatmap of 689 protein differences altered in both epilepsy and AD, segmented by proteins altered in the same direction in both disease groups, proteins altered in the opposite direction in both disease groups, and proteins with inconsistent directional changes in AD

**Fig. 3:**
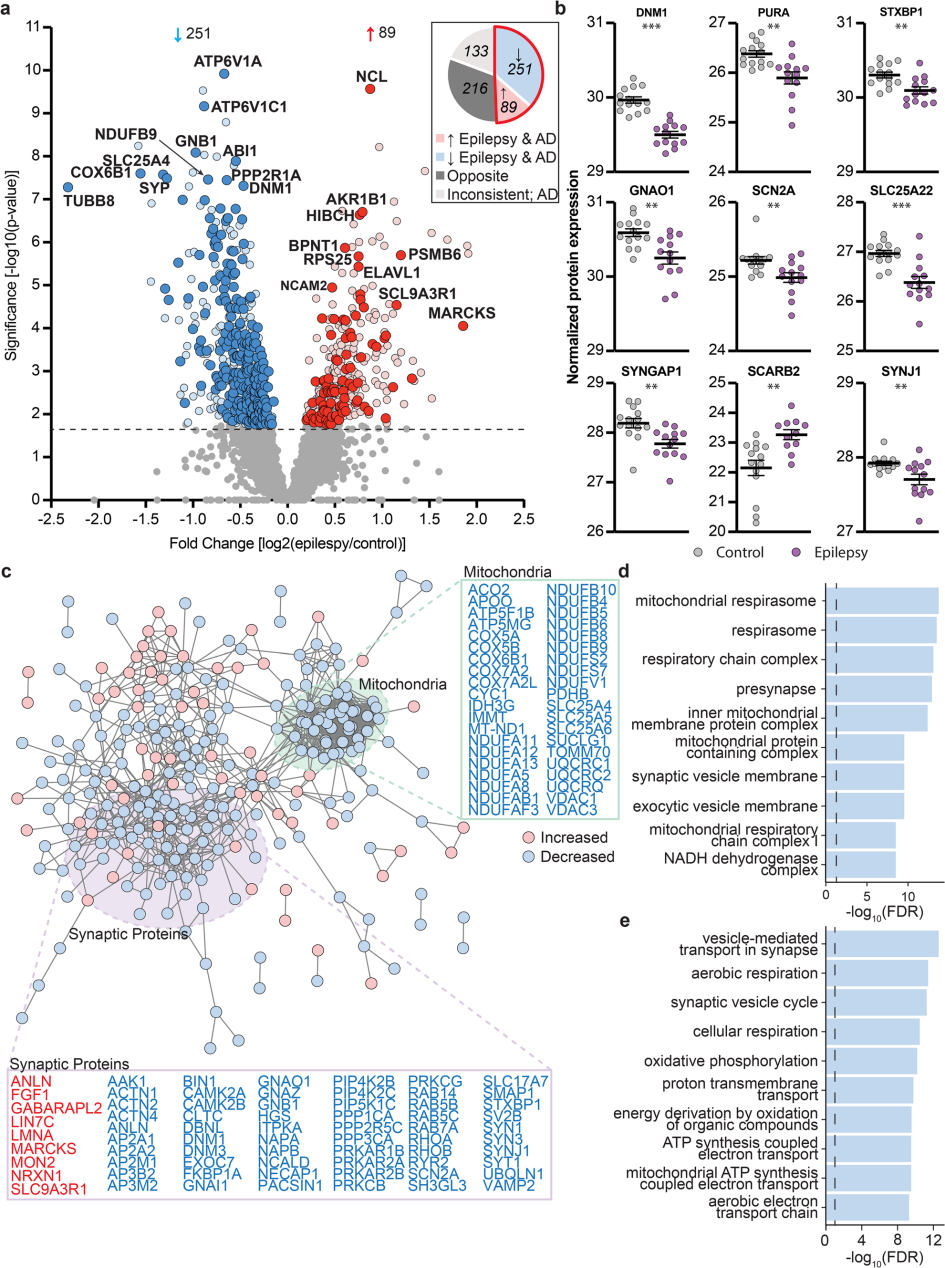
Common protein changes in epilepsy and AD. **a** Volcano plot highlighting proteins significantly altered in epilepsy. Proteins increased in both epilepsy and AD are shown in red (89 proteins) and proteins decreased in both epilepsy and AD are shown in blue (251 proteins). Proteins that were increased/decreased in epilepsy but either altered in the opposite direction in AD, unaltered in AD or inconsistently altered in AD are highlighted in pink and pale blue respectively. **b** Causative genes for human epilepsy that were altered in the same direction in both epilepsy and AD. Graphs show normalized protein expression in epilepsy and control cases obtained by LC–MS/MS analysis from [39]. **c** STRING interactome network for proteins up- and down-regulated in epilepsy. Core hubs of mitochondrial proteins and synaptic proteins identified by STRING are highlighted. **d** GO cellular component enrichments of genes down-regulated in epilepsy and **e** GO biological process enrichments for genes downregulated in epilepsy

To explore whether similar protein differences were present in human brain tissue in epilepsy and AD, we compared protein differences in the hippocampus in epilepsy (777 proteins; [39]) with protein differences in advanced AD (4743 proteins; NeuroPro database; [1]) (Fig. 1). We observed a high degree of overlap in the protein differences present in epilepsy and AD: 89% (689/777) of proteins altered in epilepsy were also significantly altered in advanced AD. Of the proteins altered in both epilepsy and AD, 49% (340/689) were altered in the same direction; 251 proteins were decreased in both epilepsy and AD and 89 proteins were increased in both epilepsy and AD (Figs. 2, 3a; Supplementary Table 1). 216 proteins were altered in the opposite direction in epilepsy and AD, while 133 proteins were altered in inconsistent directions in previous studies of advanced AD brain tissue, therefore, were unsuitable to compare in this analysis (Fig. 2; Supplementary Table 1). Nine of these common protein differences present in both epilepsy and AD were products of causative genes for human epilepsy (Fig. 3b; Supplementary Table 1). Eight of these proteins were significantly decreased in both epilepsy and AD (DNM1, PURA, STXBP1, GNAO1, SCN2A, SLC25A22, SYNGAP1, SYNJ1), while SCARB2 was increased in both epilepsy and AD. Six of the causative gene products significantly decreased in both epilepsy and AD were synaptic proteins (DNM1, PURA, SCN2A, STXBP1, SYNGAP1, SYNJ1), supporting the concept that synaptic dysfunction is an important common link between epilepsy and AD.

Proteins that were altered in the same direction in epilepsy and AD were highly interconnected (protein–protein interaction enrichment *p*-value < 1.0 × 10^–16^; Fig. 3c). Mitochondrial proteins and synaptic proteins were particularly enriched in the subgroup of proteins that were altered in the same way in both epilepsy and AD (Fig. 3c–e; Supplementary Table 8–9). 66 mitochondrial proteins were similarly altered in epilepsy and AD; of these 89% (59 proteins) were significantly decreased in both diseases (Supplementary Table 1), suggesting that widespread mitochondrial dysfunction and decreased mitochondrial proteins is a common feature of both epilepsy and AD. Decreases in proteins linked to mitochondrial respirasome (particularly respiratory chain complex I) were a common feature of both epilepsy and AD (Fig. 3d). 105 synapse proteins were altered in the same direction in epilepsy and AD. Again, the majority of these proteins (84%; 88 proteins) were decreased in both epilepsy and AD (Supplementary Table 1). Many presynaptic and postsynaptic proteins were decreased in both epilepsy and AD, indicating that synaptic dysfunction was not limited to one component of the synapse in both diseases (Supplementary Table 1). Proteins associated with synaptic vesicles were particularly decreased in both epilepsy and AD (Fig. 3e). The 89 proteins increased in both epilepsy and AD were less interconnected, resulting in no significantly enriched GO terms using our stringent search criteria.

### Opposite protein differences in epilepsy and AD

**Fig. 4:**
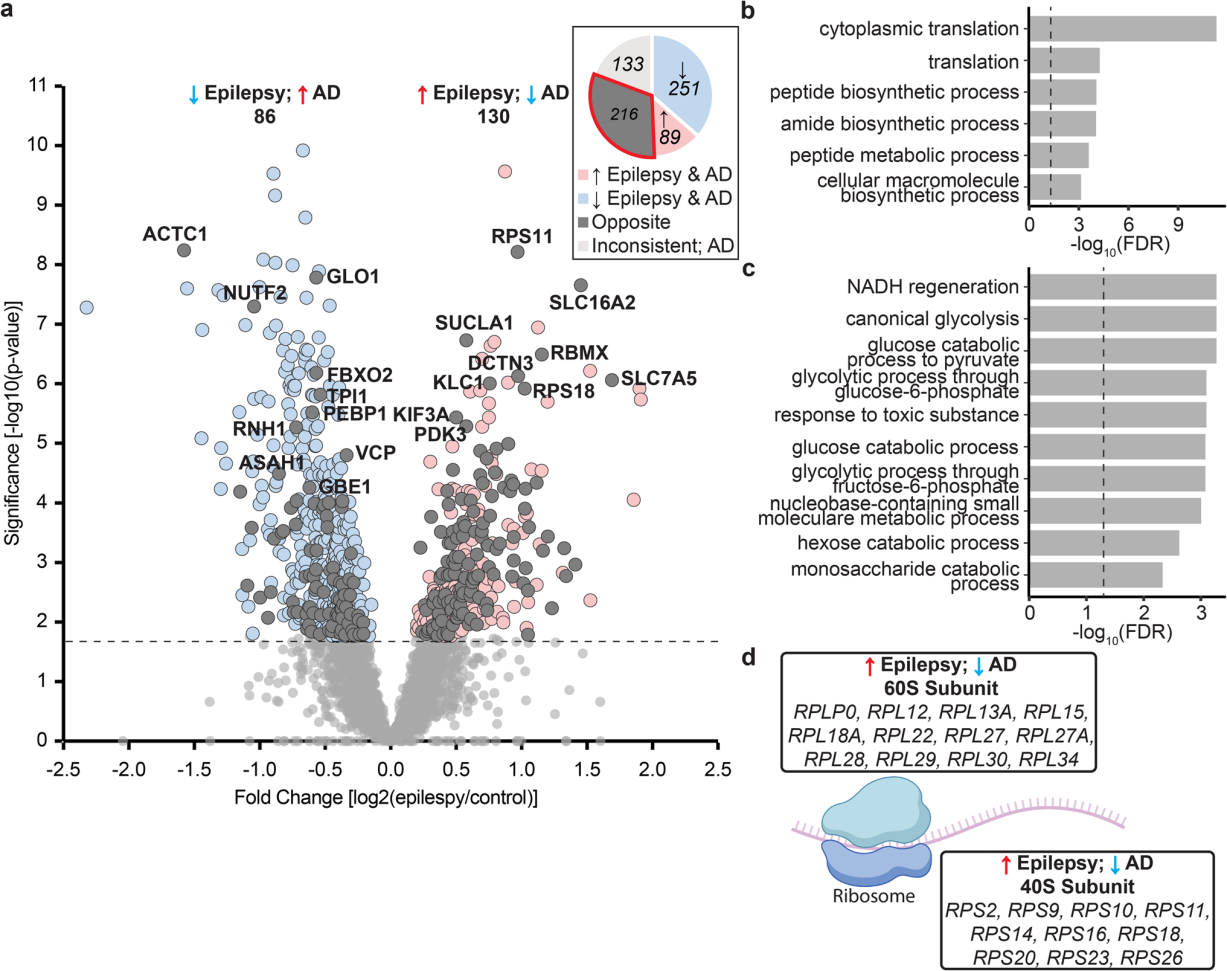
Opposite protein changes in epilepsy and AD. **a** Volcano plot shows significantly altered proteins in epilepsy that were altered in the opposite direction in AD (dark grey). 86 proteins were decreased in epilepsy and increased in AD. 130 proteins were increased in epilepsy and decreased in AD. Proteins that were increased/decreased in epilepsy but altered in either the same direction in AD, unaltered in AD or altered inconsistently in AD studies are highlighted in pink/pale blue respectively. **b** GO biological process enrichments for genes with alternative directional changes in Epilepsy (up) and AD (down). **c** GO biological process enrichments for genes with alternative directional changes in Epilepsy (down) and AD (up). **d** 60S and 40S ribosomal proteins that were increased in epilepsy but decreased in AD

216 proteins were altered in the opposite direction in epilepsy and AD: 86 proteins were decreased in epilepsy and increased in AD while 130 proteins were increased in epilepsy and decreased in AD (Figs. 2, 4a; Supplementary Table 1). This included a particularly prominent cluster of proteins linked to cytoplasmic translation that were increased in epilepsy but decreased in AD (24 proteins; Fig. 4b; Supplementary Table 10). This protein subgroup included 22 ribosome subunits that are part of both the 40S and 60S subunits (Fig. 4d; Supplementary Table 12).

### AD-associated neuropathological proteins in epilepsy

**Fig. 5:**
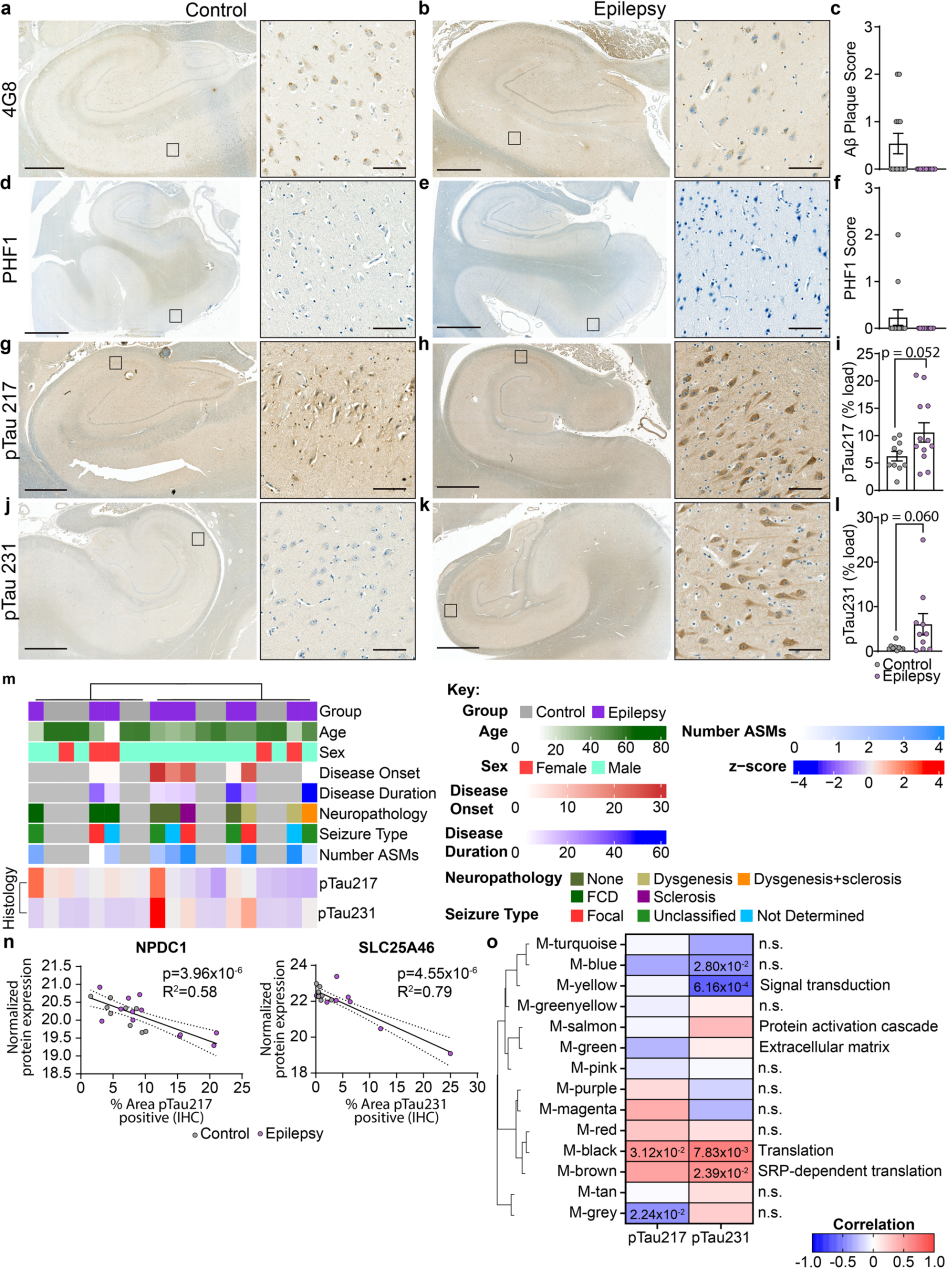
Aβ and phosphorylated tau immunostaining in epilepsy. **a**–**b** Representative images are depicted for Aβ pathology (4G8) from control (*n* = 13) and epilepsy (*n* = 13) cases at low magnification, and magnified inset from the box indicating no pathology in epilepsy cases and most of the control cases. Scale bar is 2 mm for low magnification 4G8 images. **c** Aβ pathology was semi-quantitated on a scale of 0, 1, 2, or 3. This indicated no pathology in all epilepsy cases and some pathology in five control cases. **d**–**e** Representative images are depicted for pTau396/404 pathology (PHF1) from control (*n* = 13) and epilepsy (*n* = 13) cases at low magnification, and magnified inset from the box indicating no pathology in epilepsy cases and most of the control cases. Scale bar is 4 mm for low magnification PHF1 images. **f** pTau396/404 pathology was semi-quantitated on a scale of 0, 1, 2, or 3. This indicated no pathology in all epilepsy cases and some pathology in two control cases. **g**–**h** Representative images are depicted for pTau217 from control (*n* = 10) and epilepsy (*n* = 12) cases at low magnification, and magnified inset from the box. Scale bar is 2 mm or 3 mm for low magnification control or epilepsy case images respectively. **i** There was a 1.7-fold increase in pTau217 positive area in the CA1-3 region (*p* = 0.0519), particularly for some cases. **j**–**k** Representative images are depicted for pTau231 from control (*n* = 9) and epilepsy (*n* = 10) cases at low magnification, and magnified inset from the box. Scale bar is 3 mm for low magnification images. **l** There was a 6.7-fold increase in pTau231 positive area in the CA1-3 region (*p* = 0.0600), particularly for some cases. Scale bar is 100 μm for all inset images. **m** pTau217 and pTau231 histology quantification was evaluated to determine whether tau levels clustered by clinical variables, including by group or tau levels (control *n* = 9, epilepsy *n* = 10). There was no apparent clustering by a clinical variable. **n**–**o** WGCNA of epilepsy proteomics and pTau immunohistochemistry. The top significantly correlated protein to pTau217 levels was a negative correlation to NPDC1 (*p* = 3.96 × 10^–6^, *R*^2^ = 0.58). The top significantly correlated protein to pTau231 levels was a negative correlation to SLC25A46 (*p* = 4.55 × 10^–10^, *R*^2^ = 0.79). Module trait correlation identified 5 significantly associated modules with pTau levels (*p* < 0.05). Modules were clustered by eigenprotein adjacency (relatedness to other modules) on the left. Name of module is indicated by “M-color.” *P* values are indicated for those modules with *p* < 0.05 correlation. Positive correlation is indicated in red and negative correlation in blue. Top module GO annotations are described on the right (FDR < 5%, at least 5 proteins/annotation). Several modules did not have a significant GO annotation (“n.s.”)

The high overlap of protein differences in epilepsy and AD raised the question of whether the key protein drivers of AD (Aβ and tau) could also be driving downstream brain protein differences in epilepsy. Proteomic data showed that total levels of APP and MAPT were unchanged in epilepsy [39]. Furthermore, the Aβ-specific peptide that is typically detected in proteomic studies when amyloid plaques are present (LVFFAEDVGSNK) was not identified in any epilepsy or control hippocampal sample in this cohort [39] suggesting that Aβ levels were very low in epilepsy hippocampal tissue. To confirm these proteomics findings, we performed immunohistochemistry for both Aβ and phosphorylated tau on hippocampal tissue sections obtained from the same cases as used in our previous proteomic study [39]. There was no evidence of Aβ or PHF1-immunoreactive phosphorylated tau aggregates in any epilepsy case in the hippocampus or cortex (Fig. 5a-f, Supplementary Fig. 1). In contrast, there was evidence of some Aβ and phosphorylated tau aggregates in a small number of control cases (Fig. 5c and f). We also evaluated levels of pTau217 and pTau231 as these pTau species accumulate early in AD. There was a 1.7-fold increase in pTau217 (*p* = 0.0519) and 6.7-fold increase in pTau231 (*p* = 0.0600) positive area in the CA1-3 region of epilepsy cases when compared to control cases (Fig. 5g–l). Both of these early pTau species were particularly increased in some cases in comparison to others (Fig. 5i and l). The increased pTau217 and pTau231 observed in some cases did not have morphology consistent with NFTs seen in AD, instead increased pTau staining was observed predominantly in pyramidal neurons as diffuse staining throughout the neuron soma and initial axon segment. Clinical variables such as age, sex, disease onset, disease duration, neuropathology and seizure type was not associated with pTau217 or pTau231 levels (Fig. 5m). To identify proteins and functions that correspond to increased pTau levels, WGCNA was performed. WGCNA identified 14 modules (or protein clusters) that were associated with the 2989 detected proteins (Fig. 5o, Supplementary Table 15). The top significantly correlated protein to pTau217 levels was a negative correlation to NPDC1 (*p* = 3.96 × 10^–6^, *R*^2^ = 0.58; Fig. 5n). The top significantly correlated protein to pTau231 levels was a negative correlation to SLC25A46 (*p* = 4.55 × 10^–10^, *R*^2^ = 0.79; Fig. 5n). Module trait correlation identified 5 significantly associated modules with pTau levels (*p* < 0.05). Increased pTau levels has a negative correlation to signal transduction proteins (pTau231: *p* = 6.16 × 10^–4^), and a positive correlation to translation (pTau217: *p* = 3.12 × 10^–2^, pTau231: *p* = 7.83 × 10^–3^) and SRP-dependent translation proteins (pTau231: *p* = 2.39 × 10^–2^).

The lack of plaques and NFTs in epilepsy brain tissue was surprising, given that many of the proteins altered in epilepsy are confirmed Aβ- or tau-associated proteins. For example, comparison of proteins significantly altered in epilepsy with published datasets of amyloid plaque, CAA- or NFT-enriched proteins [1] showed that 40 of the 300 confirmed amyloid plaque-enriched proteins were significantly altered in epilepsy (Supplementary Table 1). Similarly, 41 of the 192 confirmed CAA-enriched proteins and 13 of the 54 known NFT-enriched proteins were also significantly altered in epilepsy (Supplementary Table 1).

**Fig. 6:**
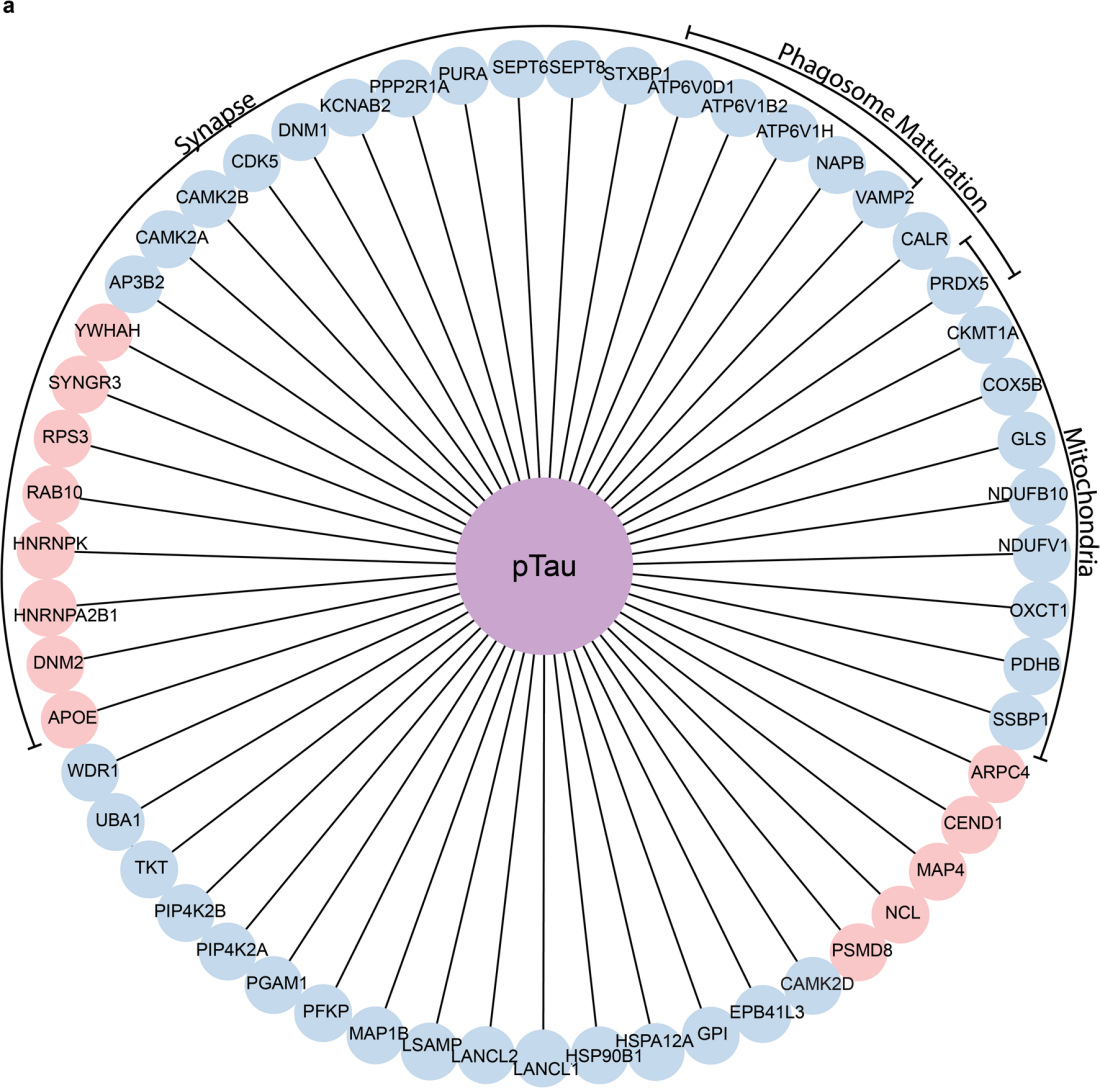
The 55 pTau interactors that are significantly altered in epilepsy. Synaptic proteins, mitochondrial proteins and proteins linked to phagosome maturation are indicated. Blue nodes are pTau interactors significantly decreased in epilepsy and red nodes are pTau interactors significantly increased in epilepsy

Intriguingly, tau was the most significant upstream regulator of proteins altered in epilepsy (Supplementary Table 6). Specifically, 118/777 proteins significantly altered in epilepsy are regulated by tau (*p* = 1.33 × 10^–57^). 82% (97/118) of these tau regulated proteins were significantly decreased in epilepsy (Supplementary Table 1), suggesting that tau could be responsible for widespread protein decreases observed in epilepsy. These tau regulated proteins have been linked to a wide variety of functions, and include many located in the synapse (44 proteins) and mitochondria (19 proteins) (Supplementary Table 1). Based on these results, we hypothesized that proteins that interact with tau would be significantly altered in epilepsy. To examine this, we compared proteins altered in epilepsy with the most comprehensive analyses of pTau and total tau interactors in human brain tissue currently available [7, 20]. Proteins altered in epilepsy were significantly enriched in proteins that interact with PHF1-immunoreactive pTau (55/125 pTau interacting proteins; *p* = 1.06 × 10^–15^) in advanced AD (Fig. 6). Most of these pTau interacting proteins were altered in the same direction in AD and epilepsy (31/55 proteins; Supplementary Table 1), providing further support for a common pathological role of pTau interacting proteins in both disease groups. pTau interacting proteins that were significantly altered in epilepsy were predominantly synaptic proteins (24 proteins), mitochondrial proteins (9 proteins) or proteins that have a role in phagosome maturation (7 proteins) (Fig. 6). Interestingly, not all key clusters of pTau interacting proteins identified in [7] were altered in epilepsy. For example, proteasome subunits were largely unaffected in epilepsy, despite being one of the most significant clusters of pTau interacting proteins previously reported [7]. Most pTau interactors were significantly decreased in epilepsy (42 proteins; Fig. 6). This included a significant cluster of mitochondrial proteins, subunits of the vATPase proton pump (ATP6V0D1, ATP6V1B2, ATP6V1H), and many synapse proteins (Fig. 6). A smaller subgroup of pTau interactors (13 proteins) were significantly increased in epilepsy, including multiple proteins associated with regulation of mRNA splicing (HNRNPA2B1, HNRNPK, NCL). While many synaptic pTau interactors were significantly decreased in epilepsy, a small subgroup of these proteins were increased in epilepsy, perhaps suggesting more complex mechanisms that underlie these protein changes beyond widespread synapse loss.

**Fig. 7:**
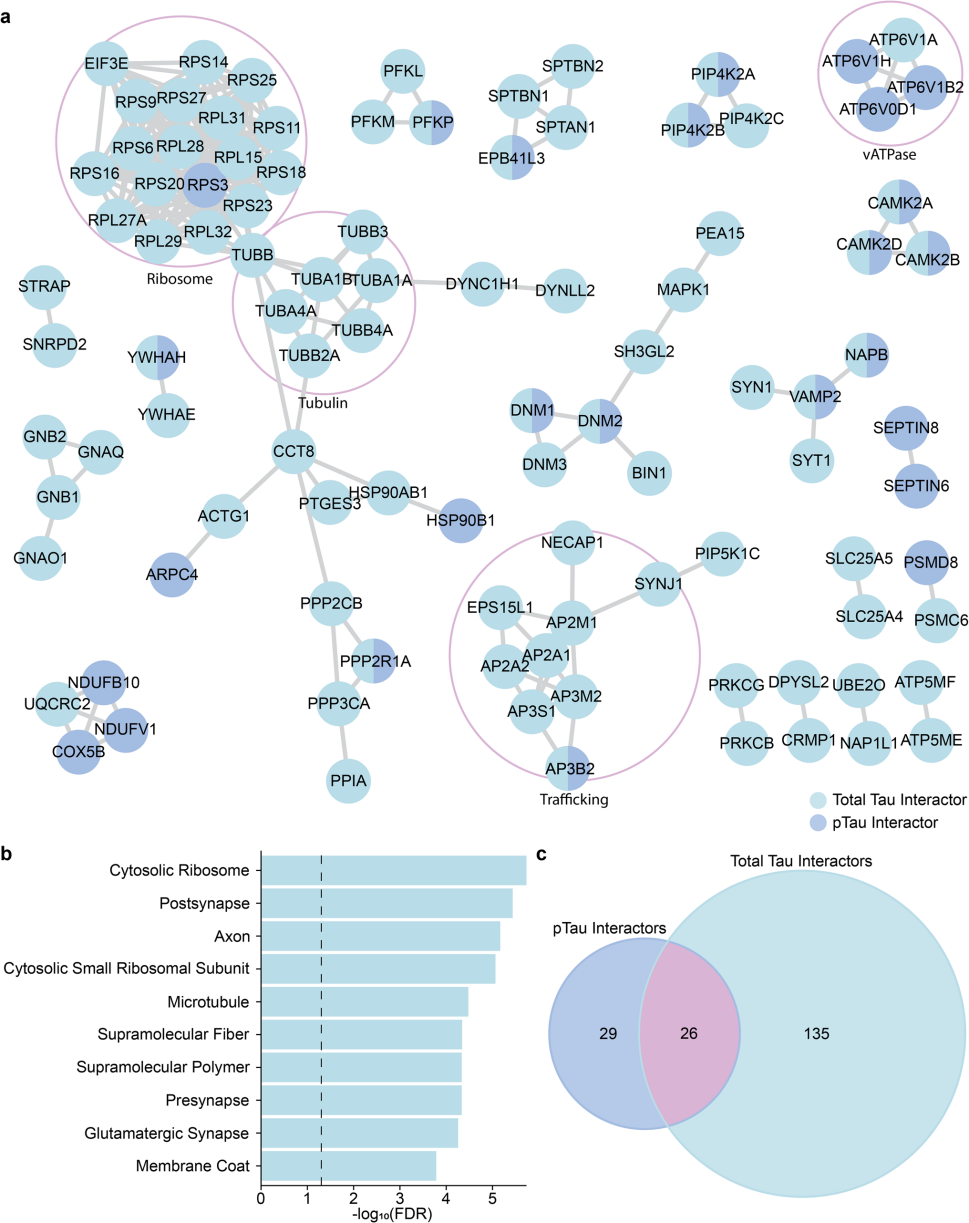
Alterations of total tau interacting proteins in epilepsy. **a** STRING network of total tau interactors that are altered in epilepsy and AD (161 proteins—101 proteins mapped in network with 60 singlets not shown). Network is limited to physical interactions only and strong confidence (0.7). **b** gene ontology cellular compartment term enrichments for the 161 proteins altered in epilepsy that are tau interactors. **c** Venn diagram of epilepsy and AD altered proteins that are pTau and/or total tau interactors

Proteins altered in epilepsy were also significantly enriched in proteins reported to interact with total tau in advanced AD (161/511 total tau interactors; *p* = 5.4 × 10^–25^). Among total tau interactors, 116/161 were decreased (Supplementary Table 1). This subgroup of total tau interactors decreased in epilepsy was significantly enriched in synaptic proteins, axon proteins and microtubule proteins (Fig. 7). A smaller cluster of 45 total tau interactors were significantly increased in epilepsy (Supplementary Table 1), notably including 16 ribosome proteins (Fig. 7; Supplementary Table 13). Together these results suggest that tau likely mediates multiple downstream protein changes in epilepsy via protein–protein interactions.

## Discussion

We identified many common protein differences in epilepsy and AD brain tissue. Our results support the hypothesis that there are common molecular mechanisms involved in epilepsy and AD, and particularly highlight common pathological changes associated with synapse and mitochondrial dysfunction. Our proteomic results suggest that tau could be a central mediator of common protein differences in epilepsy and AD. The lack of pTau neuropathological aggregates in our immunohistochemistry studies suggests that these tau-mediated effects in epilepsy can occur in the absence of advanced tau neuropathology. One explanation for these findings is that tau-regulated protein changes in epilepsy could instead be mediated by tau monomers or oligomers. This fits with the emerging body of evidence that soluble tau oligomers are likely toxic mediators in AD [10, 37], however, this requires further investigation in epilepsy as we did not directly examine tau oligomer levels in the current study. Interestingly, our previous proteomics analysis showed that global tau levels were unchanged in epilepsy in comparison to controls [39], suggesting that tau-mediated changes in epilepsy were not simply a result of altered global total tau levels. Our new findings that there were strong trends for elevated intraneuronal levels of some types of tau (pTau217 and pTau231), but not others (pTau396/404) in epilepsy suggest that only select pTau species may be altered in epilepsy. Future work examining this hypothesis is required to determine if this is the case and to characterise the tau species involved in greater detail. The two tau species we observed to be most elevated in epilepsy are also elevated in early stages of AD: pTau217 and pTau231 are increased early in the brain, pTau217 is increased in vesicles in the brain as seen by histology, as well as elevated in plasma [33, 62], suggesting that these types of tau may have an important role in early AD. The role of increased tau phosphorylation in epilepsy is an area of active investigation, with some previous studies reporting increased pTau231 [12], and increased pTau202/205, [12, 40, 45, 48, 53, 54]. Our study is the first to evaluate pTau217 in epilepsy brain tissue. Additionally, we identified functional associations that correspond to increased pTau levels by WGCNA, specifically with a negative correlation to signal transduction proteins, and a positive correlation to translation and SRP-dependent translation proteins. The effect of specific phosphorylation on cellular and synaptic functions should be investigated further in future studies in the context of both AD and epilepsy. The variability of increased pTau observed in our immunohistochemistry study adds to the increasing body of studies reporting increased pTau in some, but not all, epilepsy cases [12, 40, 45, 48, 53, 54]. The incidence of increased pTau in epilepsy requires further analysis with larger cohorts to evaluate the associated clinical variables. For example, it is possible that increased pTau is more common in some epilepsy syndromes (e.g. TLE) or when in combination with some clinical variables (e.g. long disease duration).

Our study adds support for the key role of tau in epilepsy [11, 44, 60]: our results suggest that tau may be a key upstream regulator involved in the decrease of many neuronal proteins including axon, synapse (particularly synaptic vesicle), microtubule, and mitochondrial proteins in epilepsy and that tau may mediate many of these effects via protein–protein interaction. Tau plays a physiological role in microtubule functions and other cellular functions via its other binding domains, thus tau interacts with a large number of proteins and impacts many cellular functions [4, 7, 20, 46]. Animal models reveal that tau knockdown impairs memory formation and induces anxiety-related behavior [4, 6, 55]. In AD animal models, tau reduction decreased excitotoxicity induced neuronal dysfunction [42] and in epilepsy models reduced mortality and seizure frequency [11] and behavioral impairments [51]. In an epilepsy mouse model, tau reduction prevented PI3K overactivation and decreased PTEN inhibition [51], resulting in increased mTOR signaling that increases protein translation. In our study, it was particularly interesting to see that many tau regulated proteins and tau interactors were altered in epilepsy despite no change in total tau levels in proteomic studies, suggesting that the downstream tau effects can occur regardless of bulk tissue expression levels.

In evaluating AD and epilepsy protein changes, we observed that protein differences in both disease groups were not always the same: approximately 25% of protein differences in epilepsy were altered in the opposite direction than what was observed in AD, highlighting distinct molecular mechanisms of disease. Ribosomal subunits formed the most prominent cluster of proteins that were increased in epilepsy but decreased in AD. It is well documented that ribosomal function is dysregulated in epilepsy. For example, seizures dissociate cerebral polyribosomes in animal models [61] and ribosome biogenesis is increased by chronic status epilepticus [56]. Additional studies have found widespread increases in ribosomal subunit mRNA in experimental rodent models of epilepsy and in human tissue studies, with some differences attributed to specific epilepsy syndromes and clinical variables like acute versus chronic seizure activity [15, 17, 56]. While in AD, previous AD mouse model studies indicate that with increased pTau there is decreased synthesis of ribosomal proteins [8]. In aging, there is decreased protein synthesis early in rodent models, some of which may be altered in specific brain regions and cell types differentially that could lead to selective vulnerability seen in some disease groups [47]. Ribosome protein expression is linked to the cell stress response, in which transcription is altered and can signal through p53 and mTOR pathways to influence translation, mitochondrial function, synaptic function, and cell death [38]. Further, the regulation of EIF2 signalling by the stress response and mTOR signalling have crosstalk that influences protein translation [41], though there is mTOR independent protein translation as well. The presence of increased ribosomal proteins in epilepsy suggests that pTau does not mediate the same effect on ribosomes in epilepsy as it does in AD. Future work is needed to explore the underling mechanisms and to determine if tau has a regulatory role on ribosomal protein changes in epilepsy.

Our study has some limitations. While the representative epilepsy and AD proteomes used in this study were selected because they are currently the most comprehensive datasets available, they both have limitations. The epilepsy proteomics dataset was generated from a cohort of cases encompassing a diverse range of epilepsy syndromes, therapies, and genetic backgrounds, therefore, meaning that it may not reflect proteomic differences present in select epilepsy subtypes. The AD proteomics dataset included data from multiple brain regions and, therefore, did not provide a direct comparison of hippocampus-specific protein differences. This dataset was selected as current proteomics datasets examining the AD hippocampus are limited and we have shown that AD protein differences are consistent between brain regions in AD [1], therefore, this should not affect the interpretation of our results. The epilepsy cases in this study were significantly younger than those with typical sporadic AD. However, we propose that this makes our results more robust as it increases the likelihood that the protein similarities identified in AD and epilepsy are due to disease rather than age related changes. Finally, our study is reliant on bioinformatic comparison of existing LC–MS proteomic datasets, rather than direct experimental comparison. Despite this limitation, multiple protein changes were report here as similarly altered in epilepsy and AD have been previously reported as similarly altered in human tissue or genetically linked to epilepsy, thus independently validating our results. Some examples include: Synaptophysin (SYP) is decreased in both epilepsy [39] and AD [32]; SLC17A7 (also known as VGLUT1) is decreased in select TLE cases [16] and AD [23]; DNM1L interacts with tau and accumulates in tau aggregates in AD [31] and is genetically linked to epilepsy [9]; SYN1 is decreased in AD [2] and genetically linked to seizures [29].

In conclusion, our results highlight many similarities in protein dysfunction in epilepsy and AD and provide new data for why there is a significant association between the two disease groups. If common molecular mechanisms are involved in epilepsy and AD, this raises the intriguing possibility that future AD therapeutics could be beneficial in epilepsy and vice versa. Our results increase our understanding of common mechanisms between epilepsy and AD and highlights pathways of particular importance for future studies (e.g. tau mediated changes, synapse loss, mitochondrial dysfunction). Future studies examining these potential disease drivers could have a two-fold benefit of decreasing the risk of cognitive decline in epilepsy and preventing the seizure-induced accelerated disease process in people with AD. The potential driving role of tau in epilepsy is important to understand, particularly given that recent experimental studies suggest that reduction of tau using antisense oligonucleotides could be a potential therapeutic option for epilepsy [44], an approach that was recently shown to be safe in human clinical trials for AD [36]. It will be of interest in future studies to evaluate the role of tau regulation in different epilepsy syndromes as well as in dual diagnosed AD + epilepsy cases across the disease spectrum, evaluating clinical variables like seizure frequency and clinical/subclinical epileptiform abnormalities.

### Supplementary Information

Below is the link to the electronic supplementary material.

Supplementary file1 (XLSX 577 KB)

## Data Availability

All proteomic data used in this study was obtained from prior publications that were obtained from Pubmed and from the NeuroPro database (https://neuropro.biomedical.hosting/). All data used in individual analyses are included in full in the supplementary data of this manuscript.
